# WWP1 E3 Ligase Targets LATS1 for Ubiquitin-Mediated Degradation in Breast Cancer Cells

**DOI:** 10.1371/journal.pone.0061027

**Published:** 2013-04-03

**Authors:** Benjamin Yeung, King-Ching Ho, Xiaolong Yang

**Affiliations:** 1 Department of Pathology and Molecular Medicine, Queen's University, Kingston, Ontario, Canada; 2 Cell Biology Program, Hospital for Sick Children, Toronto, Ontario, Canada; University Magna Graecia, Italy

## Abstract

The Large Tumor Suppressor 1 (LATS1) is a serine/threonine kinase and tumor suppressor found down-regulated in various human cancers. LATS1 has recently been identified as a central player of the emerging Hippo signaling pathway, which plays important roles in organ size control, tumorigenesis, and stem cell differentiation and renewal, etc. Although mounting evidence supports a role of LATS1 in tumor suppression and tumorigenesis, how LATS1 is regulated at the molecular level is not fully understood. Recently several positive regulators of LATS1 (Mst1/2, MOB1, Kibra, etc) have been identified but how LATS1 is negatively regulated is still largely unknown. We have recently identified Itch, a member of the NEDD4-like family E3 ubiquitin ligases, as a novel negative regulator of LATS1. However, whether other ubiquitin ligases modulate LATS1 stability and function is unclear. By screening many E3 ligases of the NEDD4-like family using over-expression and short-interference RNA knockdown approaches, we have identified WWP1 E3 ligase as another novel negative regulator of LATS1. We have provided *in vitro* and *in vivo* evidence that WWP1 is essential for LATS1 stability and negatively regulate LATS1 by promoting LATS1 degradation through polyubiquitination and the 26S proteasome pathway. Importantly, we also showed that degradation of LATS1 is critical in mediating WWP1-induced increased cell proliferation in breast cancer cells. Since WWP1 is an oncogene and LATS1 is a tumor suppressor gene in breast cancer, our studies provide a promising therapeutic strategy in which developed drugs targeting WWP1 cause activation of LATS1 in suppressing breast cancer cell growth.

## Introduction

LATS1 (large tumor suppressor 1) is a serine/threonine (ser/thr) kinase of the AGC kinase family and a novel tumor suppressor gene that is mutated or down-regulated in a variety of human cancers [1]. LATS1 is involved in tumorigenesis by either inducing apoptosis or negatively regulating cell proliferation, genetic stability, cell migration and metastasis [1]–[3]. Recently LATS1 has been identified as a central player of the emerging Hippo signaling pathway that was originally discovered in *Drosophila* and plays important roles in various biological processes such as tumorigenesis, organ size control, stem cell differentiation and renewal, drug resistance, and neuronal dendrite growth and tilling, etc [4]–[8]. In this pathway, ser/thr kinases and tumor suppressors Mst1/2 (mammalian homolog of *Drosophila* Hippo) and LATS1/2, and the transcriptional co-activator and oncoprotein YAP and its paralog TAZ are the core components. Mst1/2 phosphorylates and activates LATS1 and its homolog LATS2, which subsequently phosphorylates and inhibits YAP and TAZ by preventing them from translocating to the nucleus [9]–[12]. The core components Mst1/2-LATS1/2-YAP/TAZ also interact with upstream (e.g. Fat4, Mer, RASSF1A, Kibra, etc.) and downstream signaling molecules (e.g. CTGF, Cyr61, Axl, etc.) in regulating various biological functions (for review, see [7], [13]).

Despite the critical role of LATS1 in the Hippo pathway, how LATS1 is regulated at the protein level is largely unknown (for review, see [1]). Recently several positive regulators of LATS1 such as Mst1/2, hMOB1, and Kibra have been identified [9], [14], [15]. However, how LATS1 is negatively regulated is largely unknown. Importantly, we have identified the E3 ubiquitin ligase Itch, a member of the NEDD4-like ubiquitin ligase family, as the first negative regulator of LATS1 [16]. However, the NEDD4-like family consists of nine members (i.e. Itch, NEDD4, NEDD4-2, WWP1, WWP2, Smurf1, Smurf2, NEDL1, and NEDL2). Whether other members of the same NEDD4-like family are also involved in the regulation of LATS1 under different cellular context is unknown.

WWP1 (WW domain containing E3 ubiquitin protein ligase 1) is a member of the NEDD4-like family of HECT ubiquitin ligase and plays important roles in a diverse variety of biochemical and cellular processes, such as protein degradation, transcriptional regulation, cell proliferation and differentiation, apoptosis, and senescence [17], [18]. WWP1 contains a C2 calcium binding domain, four WW domains, and a HECT domain for transferring ubiquitin to target proteins. WWP1 regulates various biological functions mostly by interacting target proteins with its C2 or WW domains and directing them for degradation by the 26S proteasome pathway via polyubiquitination. So far, several WWP1 substrates including p27, KLF2, Smad2-6, ErbB4, p63 etc. have been identified (for review see [18]). It has been shown that WWP1 can regulate senescence, TGFβ signaling and bone differentiation and metastasis, and EGF signaling by causing degradation of p27, Smad7, and EGFR/ErbB2/ErbB4, respectively [19]–[22]. Importantly WWP1 has been identified as an oncogene. Amplification and over-expression of WWP1 has been found in breast and prostate cancers [23]–[26].

Recently, mounting evidence suggests that degradation of tumor suppressors by E3 ubiquitin ligases may play important roles in tumorigenesis. For example, degradation of tumor suppressors p53 by Mdm2 and Pirh2 ligases [27], [28], PTEN by NEDD4 [29], p73 and p63 by Itch [30], [31], is crucial for cancer development and progression. Therefore, targeting oncogenic E3 ubiquitin ligases to activate tumor suppressors (i.e. p53) to kill cancer cells has become one of the most important strategies for cancer therapy [32]. Identification of ubiquitin ligases that regulate tumor suppressors will be very useful for developing novel therapeutic drugs for cancer treatment in the future. However, while many ubiquitin ligases have been identified for a limited number of tumor suppressors, the ubiquitin ligases regulating many other tumor suppressors remain largely unknown. In this study, we have shown that WWP1 E3 ubiquitin ligase regulates cell proliferation by targeting tumor suppressor LATS1 through ubiquitin-mediated protein degradation.

## Materials and Methods

### Plasmid construction

The LATS1-FLAG, LATS1-Y376A/Y559A-FLAG, LATS1-Y376A/Y559A-myc, WWP1-FLAG, and WWP1-myc plasmids were constructed as previously described [16]. KOD Hot Start DNA polymerase (Novagen) was used for all PCRs. For construction of NEDD4-myc, Smurf1-myc, Smurf2-myc, and WWP1-myc plasmids, open reading frames of each gene were amplified by PCR using EST plasmids purchased from Open Biosystems as templates using the following primers:

1. BamHI-NEDD4-1F: 5′-CTAGGATCCATGGCAACTTGCGCGGTGG-3′

2. NotI-NEDD4-1R: 5′-GAATATTATGCGGCCGCTCTAATCAACTCCATCAAAG-3′

3. Bgl2-Smurf1-F: 5′-GA AGA TCT ATG TCG AAC CCC GGG ACC CG-3′

4. NotI-Smurf1-R: 5′-G TAA TCA TGC GGC CGC TCA CTC CAC TGC AAA GCC AC-3′

5. EcoRI-Smurf2-F: 5′-AT GAA TTC ATG TCT AAC CCC GGA GGC C-3′

6. NotI-Smurf2-R: 5′-G TAA TCA TGC GGC CGC TCA TTC CAC AGC AAA TCC AC-3′

7. Bgl2-WWP1-F: 5′-GA AGA TCT ATG GCC ACT GCT TCA CCA AGG-3′

8. NotI-WWP1-R: 5′-G TAA TCA TGC GGC CGC TCA TTC TTG TCC AAA TCC CTC-3′

The PCR products were digested by BamHI/NotI, Bgl2/NotI, or EcoRI/NotI, and ligated into pcDNA3.1-hygro-myc vector digested with the same enzymes.

For making WWP1-C890A, site directed mutagenesis method was used as previously described [9]. For making WWP1 mutant lacking WW domains (WWP1-ΔWW-myc), over-lapping PCR method was used. For the first round of PCR, two PCR reactions amplifying 5′ and 3′ ends surrounding the deletion region containing WW domains (aa. 350–530) were carried out using WWP1 EST cDNA plasmids as templates and the following primers: PCR#1: Bgl2-WWP1-F (forward): see sequence above; WWP1-349-R (reverse): 5′- TTC TGT GTT GGC ATT CCC AGA-3′; PCR#2: WWP1-1-349BR530-F (forward): 5′ - TCT GGG AAT GCC AAC ACA GAA GGG AAG TCA TCT GTA ACT AAA - 3′; NotI-WWP1-R (reverse): see sequence above. For the second round of PCR, 50 ng each of PCR#1 and PCR#2 products from the first round PCR were mixed and run for the following program: 1 cycle at 94°C for 2 min; 5 cycles at 94°C for 15 seconds, 60°C for 30 seconds, 68°C for 90 seconds. After adding Bgl2-WWP1-F and NotI-WWP1-R1 primers, the following program was run: 25 cycles for 15 seconds, 60°C for 30 seconds, 68°C for 90 seconds, and 1 cycle at 70°C for 2 min. The PCR product was digested with Bgl2/Not I and subcloned into BamHI/NotI sites of pcDNA3.1-hygro-myc vector.

For lentiviral production, WWP1 or WWP1-C890A cDNA was first amplified by PCR using WWP1 or WWP1-C890A cDNA plasmids as templates, digested by Bgl2/Not I, and subsequently cloned into the BamHI/Not I site of pcDNA3.1-hygro-3×FLAG [9]. The FLAG-tagged WWP1 or WWP1-C890A cDNA was subsequently cut out from the vector by PmeI and subcloned into the PmeI site of the WPI lentiviral vector.

### Cell culture and transfection

MCF10A cells were maintained in Dulbecco's modified Eagle's medium and F12 (DMEM-F12) medium supplemented 5% horse serum, 20 ng/ml hEGF, 0.5 µg/ml hydrocortisone, 10 µg/ml insulin, 100 ng/ml cholera toxin, 2.5 mM L-glutamine, and 1% penicillin-streptomycin). All cells were cultured at 37°C in a 5% CO_2_ atmosphere. COS7, HEK293T, and MCF7 cells were maintained in DMEM containing 10% fetal bovine serum (FBS) and 1% penicillin-streptomycin (P/S). T47D cells were grown in RPMI-1640 media containing 10% FBS and 1% P/S. For transient transfection of COS7 and HEK293T cells, Lipofectamine 2000 (Invitrogen, Burlington, Canada) was used according to the manufacturer's instructions.

### Antibodies, western blotting and co-immunoprecipitation (Co-IP)

Mouse monoclonal antibodies to Myc (Roche), FLAG (Sigma), WWP1 (Novus Biologicals), and Itch (BD Biosciences) were clones 9E10, M2, 1A7, and 32/Itch, respectively. Rabbit polyclonal antibody to Myc (A14) and Smurf1 was purchased from Santa Cruz and Cell Signaling, respectively, whereas rabbit monoclonal antibodies to Myc (71D10) and Smurf2 were purchased from Cell Signaling and Epitomics, respectively. Rabbit α-LATS1 (Y03) polyclonal antibody is as described [9]. For interaction of LATS1-FLAG with WWP1-myc, transfected COS7 cell lysates were harvested and 1 mg of each lysate sample was immunoprecipitated using 2 ug of mouse anti-Myc 9E10 monoclonal antibody (Roche). Immunoprecipitated proteins were then detected by western blotting using mouse anti-FLAG (M2) monoclonal antibody (Sigma). For interaction between endogenous LATS1 and WWP1, 1 mg of HEK293T cell lysates were immunoprecipitated with either pre-immune serum or rabbit anti-LATS1 polyclonal antibody (Y03). Immunoprecipitated proteins were then detected by western blotting with a mouse anti-WWP1 monoclonal antibody (Novus Biologicals).

### GST fusion protein production and pull-down assays

GST and GST fusion proteins were produced and purified as previously described [9]. For GST pull-down assays, about 100 µg of protein lysate expressing FLAG tagged LATS1 or LATS1-Y376A/Y559A was mixed with 10 µg of GST (control), WWP1-GST, WWP1-ΔWW-GST, or WW domain GST fusion proteins on beads and incubated at 4°C with rotating for 2 h. The beads were then washed four times with lysis buffer (50 mM Tris-HCl pH 7.4, 150 mM NaCl, 1 mM EDTA and 1.0% Nonidet P-40), resuspended in 2×SDS sample buffer, boiled, and centrifuged. The resulting supernatants were subjected to SDS-PAGE and western blot analysis using mouse monoclonal anti-FLAG antibody (M2, Sigma).

### RNA extraction and qRT-PCR

RNA extraction and qRT-PCR were performed as previously described [4], [9]. The following primers were used for qPCR: 1. WWP1: Forward, 5′-GAG GAT GAT TCT CCA TTA ACA GTG-3′; Reverse, 5′-GAG GAT GAT TCT CCA TTA ACA GTG-3′. 2. LATS1: Forward, 5′-CAG CTG CCA GAC CTA TTA ATG C-3′; Reverse, 5′-AAT GAT AGG CCA CAC TTT CTC C-3′.

### Cycloheximide (CHX) chase measurements of LATS1 half-life

COS7 cells were transiently transfected with either LATS1-FLAG alone or together with WWP1-myc using Lipofectamine 2000. At 16 hours post-transfection, culture medium was replaced by DMEM supplemented with 10% FBS, 1% penicillin-streptomycin and CHX (20 µg/ml). Cells were lysed at 0, 4, 8, 12 and 24 hours following CHX treatment and the resulting lysates were analyzed by western blotting using mouse anti-FLAG (M2, Sigma) and rabbit anti-Myc (71D10, Cell Signaling) monoclonal antibodies.

### In vivo and in vitro ubiquitination assays

For *in vivo* ubiquitination assays, HEK293T cells were transiently transfected with plasmids expressing ubiquitin-HA and LATS1-FLAG alone or together with WWP1-myc or WWP1-C890A-myc using Lipofectamine 2000. At 5 h post-transfection, cells were treated with 5 µM MG132 for an additional 24 hours prior to being lysed by modified RIPA buffer (2 mM Tris-HCl pH 7.5, 5 mM EDTA, 150 mM NaCl, 1% NP40). 20 µl of Protein A beads and 1 mg of each lysate sample was precleared and immunoprecipitated by mouse anti-FLAG M2 monoclonal antibody. The ubiquitinated-LATS1 was detected by western blotting using rabbit anti-HA polyclonal antibody (Epitomics).

For *in vitro* ubiquitination, 1 mg of protein lysate extracted from cells expressing LATS1-myc was immunoprecipitated by rabbit monoclonal anti-Myc (71D10) antibody (Cell Signaling) bound on protein A beads (Roche), washed 4 times with modified RIPA buffer, followed by incubation in 30 µl of ubiquitination conjugation reaction buffer [2 mM ATP, 0.5 µg Ubiquitin-FLAG, 0.1 µM ubiquitin activating enzyme (E1), and 0.5 µM ubiquitin conjugating enzyme (E2, UbcH7, Boston Biochem) in the absence or presence of WWP1-GST (E3) or WWP1-C830A-GST (ligase-dead) at 30°C for 90 min. As a negative control, WWP1-GST alone was also incubated with ubiquitin conjugation reaction buffer. The beads containing ubiquitinated LATS1 were spun down, washed 4 times with modified RIPA buffer, resuspended in 2×SDS sample buffer (200 mM Tris-HCl, pH 6.8, 200 mM DTT, 20% glycerol, 4%SDS, and 0.02% bromophenol blue), boiled at 100°C for 5 min, and subjected to western blot analysis using anti-FLAG (M2, Sigma) antibody.

### Knockdown of ubiquitin ligase by short interference (si) RNA

The ON-TARGET*plus* siRNA duplex targeting Smurf 1, Smurf2, Itch, WWP1, or NEDD4 mRNA and a negative control siRNA with scrambled sequence absent in the human genome were purchased from Dharmacon RNA Technologies. Pools of four double-stranded siRNA duplexes targeting different regions of each ubiquitin ligase mRNA were used. For cell transfection, 2×10^5^ MCF7 or T47D cells were seeded into each well of a 6-well plate and subsequently transfected with a concentration of 100 nM siRNA using Lipofectamine 2000 (Invitrogen). Two days post transfection, proteins were extracted, and the levels of each E3 ubiquitin ligase and LATS1 were examined by western blot using β-actin as internal loading control.

### Lentiviral production and establishment of stable cell lines

Lentiviral production and purification were performed as previously described [16]. MCF10A cells stably expressing WPI (vector control) or WWP1 were established by infection of the cells with either the WPI or WWP1 lentivirus. For stable shRNA knockdown of WWP1, MCF7 breast cancer cells were infected with lentivirus expressing pGIPZ (vector control), shWWP1-1, or shWWP1-2. Since pGIPZ vector expresses a puromycin-resistant gene, cells with shRNA expression were selected by incubation in 2 µg/ml of puromycin following infection. Expression of WWP1 was examined by western blot using mouse anti-WWP1 monoclonal antibody (Novus Biologicals). For shWWP1 rescue experiment, MCF7 expressing shWWP1-2, which targets untranslated region of WWP1 mRNA, were infected with lentivirus expressing WWP1 or WWP1-C890A. For LATS1 rescue experiments, the MCF7 cells were simultaneously infected with both shWWP1-1 and shLATS1 lentiviruses, followed by puromycin selection.

### Cell proliferation analysis and colony forming assays

For cell proliferation analysis, triplicate of 2×10^4^ cells were seeded into each well of 24-well plate. Cell numbers were counted every day for 6 days. The mean and standard deviation (SD) of three samples are calculated for each day. All of the experiments were repeated at least three times. Colony forming assays were performed as described previously [16].

## Results

### Screen for E3 ubiquitin ligases regulating LATS1 stability

**Figure 1:**
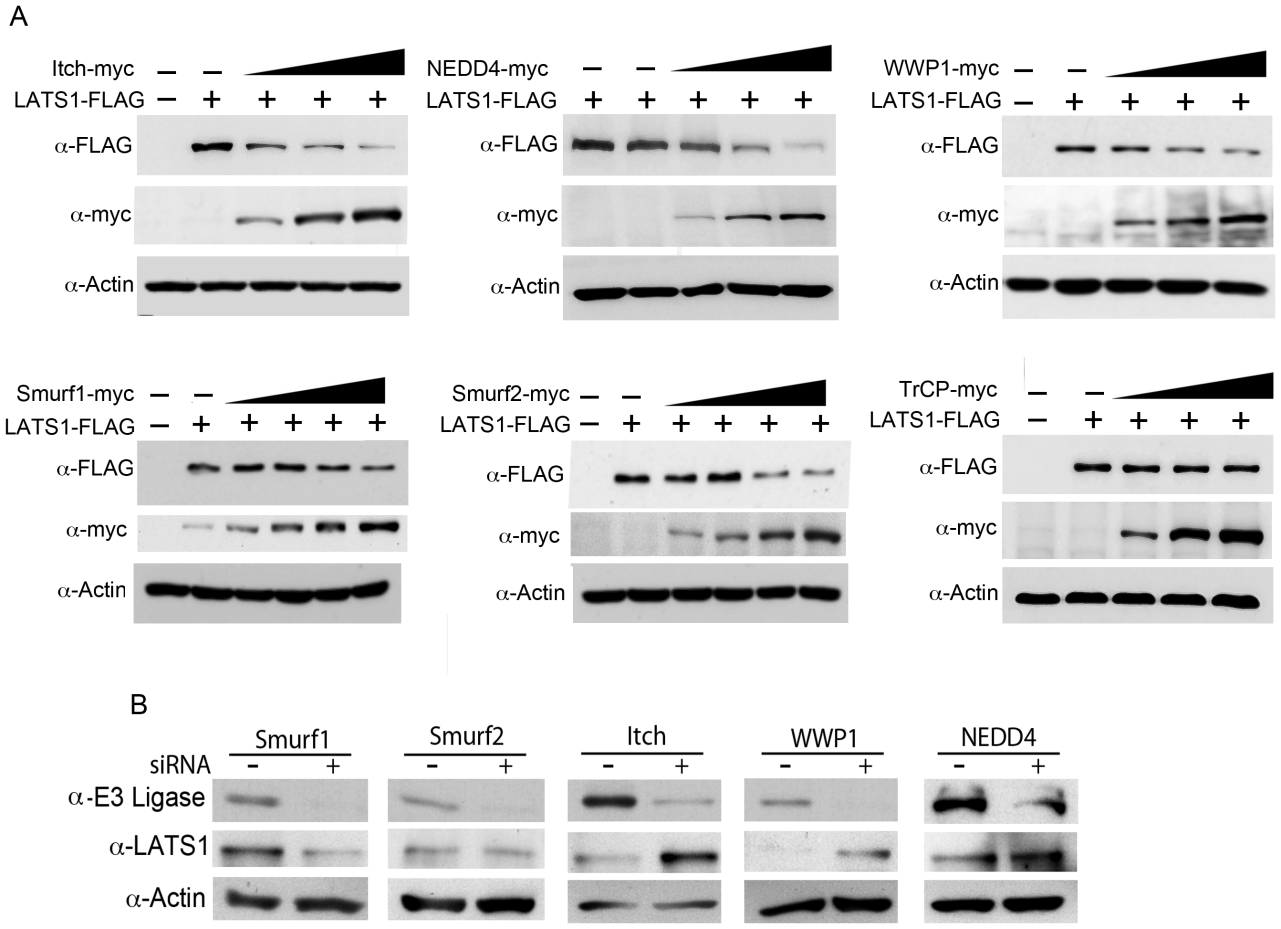
Effect of NEDD4-like family E3 ubiquitin ligases on LATS1 stability. A) Dose-dependent degradation of LATS1 by E3 ubiquitin ligases. LATS1-FLAG plasmids were transfected alone (0.2 µg) or together with increasing amounts [0.05, 0.1, 0.2, 0.4 µg] of WWP1 plasmids into COS7 cells. The levels of LATS1-FLAG and WWP1-myc were detected by western blotting using anti-FLAG and anti-Myc antibodies, respectively. B) Knockdown of NEDD4-like family E3 ubiquitin ligases on LATS1 stability. About 100 nM of siRNA targeting scrambled RNA (negative control) or each E3 ligase was transfected into MCF7 or T47D cells in a 6-well plate. Two days after transfection, proteins were extracted and subjected to western blot analysis of each E3 ligase, LATS1, and β-actin (internal loading control).

To understand whether LATS1 stability is also regulated by other members of the NEDD4-like family of E3 ligases, we selected 4 ubiquitin ligases including NEDD4, WWP1, Smurf1 and Smurf2 that have been shown to be involved in the development of human cancers [33], [34]. We also used Itch as a positive control. Although similar to Itch, over-expression of any of the E3 ligases including NEDD4, WWP1, Smurf1, and Smurf2 causes dose-dependent reduction of LATS1 (Fig. 1A), only siRNA knockdown of WWP1 like that of Itch caused significant increases on endogenous LATS1 levels in breast cancer cells (Fig. 1B), suggesting that Itch and WWP1 are essential E3 ubiquitin ligases regulating stability of LATS1. As a negative control, over-expression of TrCP1, which is not NEDD4-like family member, has no effect on LATS1 (Fig. 1A). Since down-regulation of LATS1 and over-expression of WWP1 was shown to be involved in the development of breast cancer [25], [35], we further explored the physical and functional interaction of WWP1 and LATS1.

### WWP1 interacts with LATS1 in vivo and in vitro

**Figure 2:**
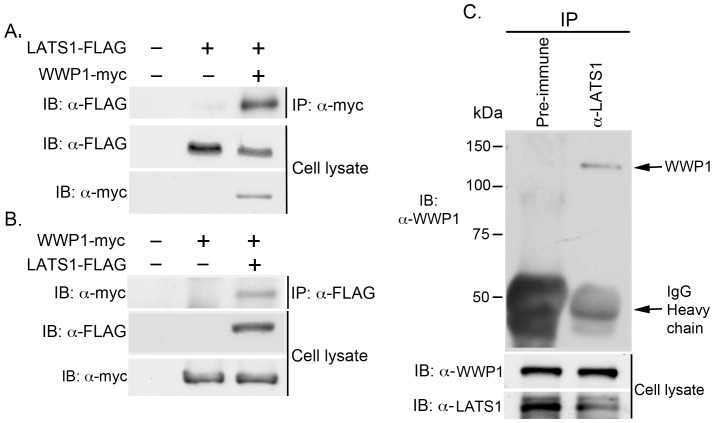
Interaction of LATS1 and WWP1 *in vivo*. A, B) Co-IP of ectopically expressed LATS1 and WWP1 *in vivo*. COS7 cell lysates expressing either LATS1-FLAG (A) or WWP1-myc (B) alone or together with WWP1-myc (A) or LATS1-FLAG (B) were immunoprecipitated with anti-Myc (A) or anti-FLAG (B) antibody, followed by western blotting with anti-FLAG antibody (A) or anti-Myc (B) antibody, respectively. Expression of LATS1 levels in cell lysate was also examined by western blot (A, B). C) Co-IP of endogenous LATS1 and WWP1 *in vivo*. About 1 mg of cell lysates from HEK293T were immunoprecipitated with either control pre-immune serum or rabbit anti-LATS1 (Y03) polyclonal antibody, followed by western blotting with anti-WWP1 antibody. Expression of endogenous WWP1 and LATS1 is also shown.

We first used Co-IP assays to examine whether WWP1 interacts with LATS1 *in vivo*. FLAG-tagged LATS1 was either transfected alone or together with myc-tagged WWP1 into COS7 cells. When WWP1-myc was precipitated from protein lysate with anti-Myc antibody, LATS1-FLAG was detected in the WWP1-myc immune complex only when both LATS1-FLAG and WWP1-myc were co-transfected into cells (Fig. 2A). Vice versa, when WWP1-myc was transfected alone or together with LATS1-FLAG into COS7 cells, WWP1-myc was detected in the LATS1-FLAG immune complex only when both WWP1-myc and LATS1-FLAG were co-transfected into cells (Fig. 2B). To exclude the possibility that LATS1-WWP1 interaction is due to their high level of protein expression in the cells, we also examined the interaction of endogenous LATS1 with WWP1 in HEK293T cells. Significantly, endogenous WWP1 was detected only in the immune complex precipitated with anti-LATS1 polyclonal antibody rather than its pre-immune serum (Fig. 2C).

**Figure 3:**
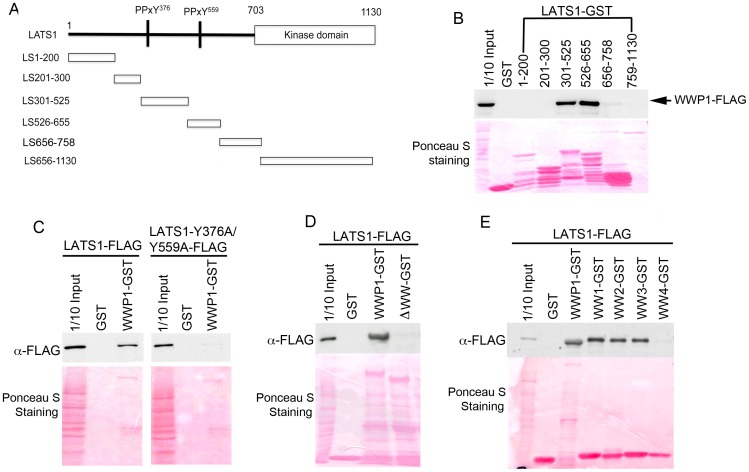
Interaction of LATS1 and WWP1 *in vitro*. A) Diagram of LATS1 deletion. B-E) GST pulldown analysis of functional domains responsible for the interaction of LATS1 and WWP1. About 100 µg of protein lysates from COS7 cells expressing FLAG-tagged LATS1 or its mutants (C–E), or WWP1 (B) was mixed with 10 µg of GST (control) or GST fusion proteins (LATS1 deletions or WWP1 mutants) on beads. The beads were washed and the binding proteins were eluted and subjected to western blot using anti-FLAG antibody. 1/10 input (10 µg) represents 1/10 of protein lysate (100 µg) used for GST pulldown. Ponceau S was used to stain fusion proteins on the membrane. B) Functional domains of LATS1 interacting with WWP1. Lysate: WWP1-FLAG; LATS1 deletion GST fusion proteins. C) Interaction of LATS1-PPxY mutants with WWP1 *in vitro*. Lysate: LATS1-FLAG (wild-type) or LATS1 with double PPxY mutation (LATS1-Y376A/Y559A-FLAG). D) Functional domains of WWP1 interacting with LATS1 *in vitro*. ΔWW-GST: WWP1 lacking all of 4 WW domains. E) Interaction of LATS1 with each WW domain of WWP1 *in vitro*. WW1-4 GST: first to fourth WW domain GST fusion protein.

We also used GST-pull down assay to examine whether LATS1 directly interacts with WWP1 *in vitro*. LATS1-FLAG was able to bind strongly to WWP1-GST *in vitro* (Fig. 3C). To understand the molecular mechanism of LATS1-WWP1 interaction, we further mapped the domains in both LATS1 and WWP1 responsible for their interactions. First, we performed a GST-pull down assay using cell lysate expressing WWP1-FLAG and GST fusion proteins with a series of LATS1 deletions (Fig. 3A). WWP1-FLAG was found to specifically bind to LATS1-301-525-GST and LATS1-526-655-GST (Fig. 3B). WWP1 belongs to Group I of the WW domain proteins that bind their partners through their WW domains to PPxY (P, proline; X, any amino acid, Y, tyrosine) or PPRXXP or PPPPP motifs in their binding substrate [36], [37]. There are two PPXY motifs in LATS1, PPPY^376^ and PPPY^559^, which are located in the WWP1 binding regions aa. 301–525 and aa. 526–655 of LATS1 (Fig. 3B), respectively (Fig. 3A). Therefore, we tested whether these two PPXY motifs are responsible for the interaction of LATS1 with WWP1. Mutation of “Y” to “A” (alanine) for both motifs (LATS1-Y376A/Y559A) completely abolished its binding to WWP1-GST (Fig. 3C), suggesting that both PPxY^376^ and PPxY^559^ motifs in LATS1 are responsible for its interaction with YAP.

Previous studies have shown that WWP1 usually interacts with PPxY motifs of its target protein using its WW domains [18]. To determine whether the WW domains in WWP1 are responsible for its binding to LATS1, we created a WWP1 mutant with all 4 WW domains deleted (WWP1-ΔWW-GST). As expected, deletion of WW domains in WWP1 completely abrogated its binding to LATS1 (Fig. 3D). To determine which WW domain in WWP1 is responsible for its binding to LATS1, we further made constructs expressing each of the 4 WW domains. Although similar amounts of GST fusion protein were used, WW domains 1–3 GST fusion proteins (WW1-GST, WW2-GST, and WW3-GST) rather than WW4-GST or GST alone bind to LATS1-FLAG (Fig. 3E). In conclusion, our results suggest that LATS1 binds to the 1–3 WW domains of WWP1 through its PPxY^376^ and PPxY^559^ motifs.

### WWP1 specifically targets LATS1 for ubiquitin-mediated degradation in breast cancer cells

**Figure 4:**
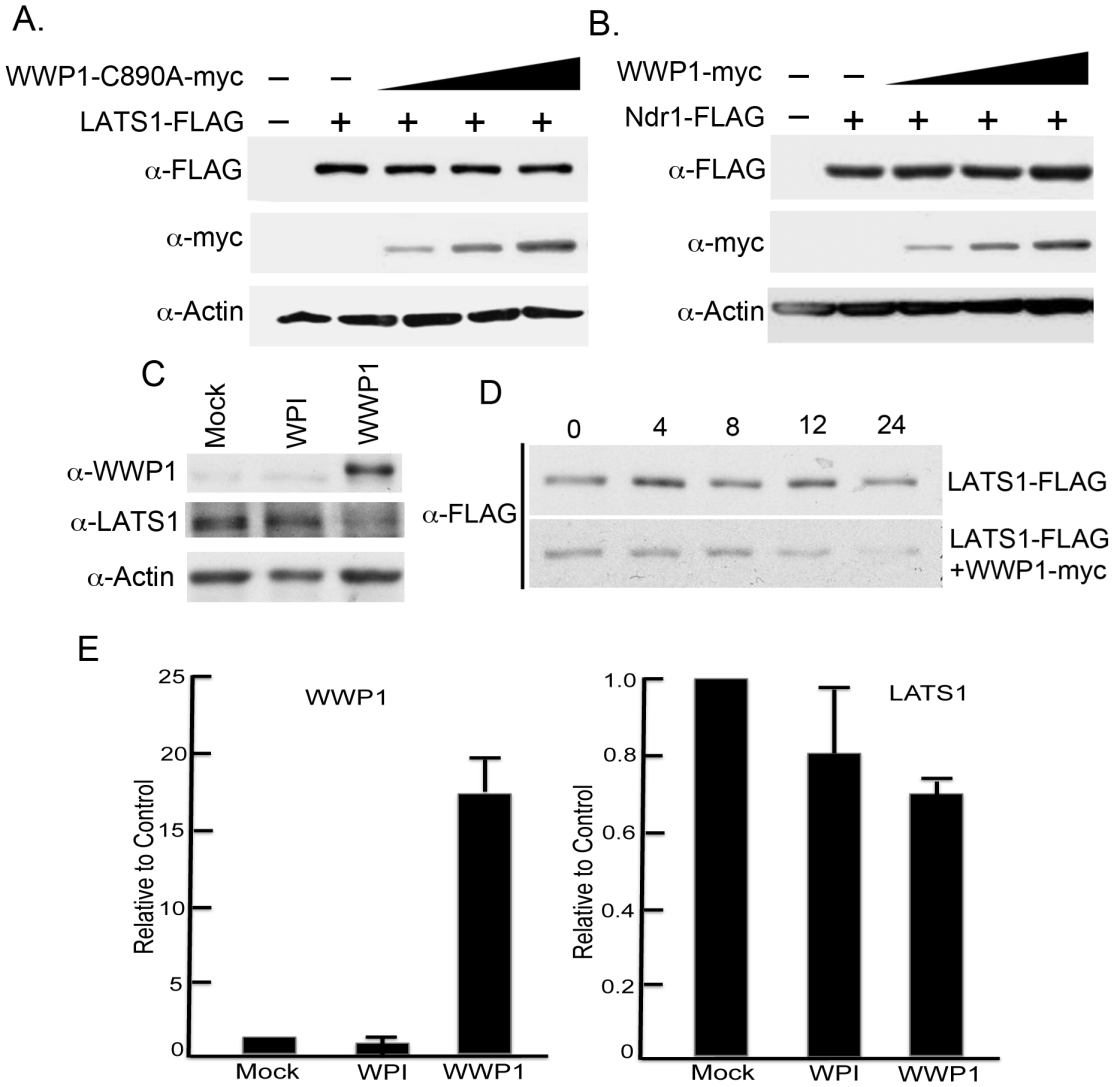
Regulation of LATS1 protein stability by WWP1. A) Ligase-dependent degradation of LATS1 by WWP1. Increasing amounts of ligase-dead WWP1 (WWP1-C890A-myc) was co-transfected with equal amounts of LATS1 plasmids. B) Inability of WWP1 to degrade LATS1 homolog Ndr1 lacking PPxY motifs. C) Degradation of endogenous LATS1 by WWP1 in MCF10A mammary cells. MCF10A cells were mock infected or infected with WPI (vector) or WWP1-WPI lentivirus. After establishment of stable lines, endogenous WWP1, LATS1, or β-actin was detected by western blot. D) Measurement of WWP1 half-life by CHX chase assays. LATS1-FLAG alone or together with WWP1-myc were transfected into COS7 cells, followed by treatment with CHX to inhibit protein synthesis. At the indicated time, cells are harvested and analyzed for LATS1 level using anti-FLAG antibody. E) Expression of LATS1 mRNA after WWP1 over-expression. About 0.1 µg of RNAs was used for one-step qRT-PCR (Invitrogen) analysis using rRNA as controls. Values represent mean and standard deviation of fold increase of mRNA levels in WPI or WWP1-expressing MCF10A cells relative to those in mock-infected MCF10A cells.

**Figure 5:**
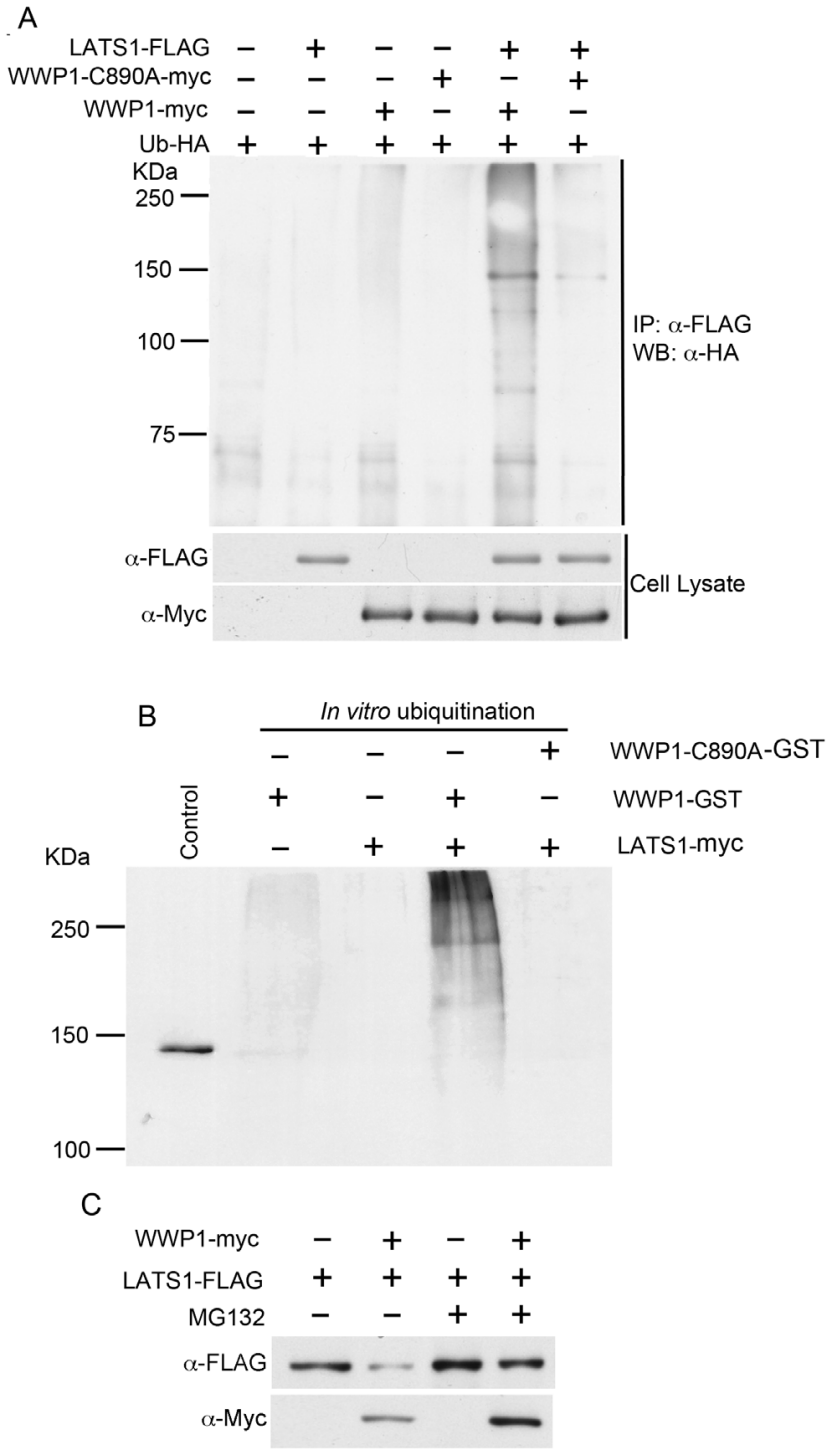
WWP1 promotes LATS1 degradation by polyubiquitination and the proteasome pathway. A) Ubiquitination of LATS1 by WWP1 *in vivo*. Ubiquitin-HA and different combination of WWP1-myc, WWP1-ligase-dead mutant (WWP1-C890A-myc) and LATS1-FLAG were transfected into HEK293T cells. Ubiquitinated LATS1 was detected by immunoprecipitation of LATS1 with anti-FLAG antibody, followed by detection of ubiquitin using anti-HA antibody. B) Ubiquitination of LATS1 by WWP1 *in vitro*. Immunoprecipitated LATS1-myc or LATS1-Y376A/Y559A-myc on beads was used as a substrate in an ubiquitination assay with a ligase buffer containing E1, E2, Ubiquitin-FLAG, ATP, and WWP1-GST or WWP1-C890A-GST. After the reaction, beads containing LATS1-myc were washed extensively with modified RIPA buffer, followed by western blot analysis using anti-FLAG antibody. Cell lysate expressing FLAG-tagged LATS1 was used as positive control (lane 1). C) Inhibition of WWP1-mediated LATS1 degradation by proteasome inhibitor MG132. COS7 cells transfected with either LATS1-FLAG alone or together with WWP1-myc were treated with either DMSO (control) or proteasome inhibitor (MG132), followed by examination of LATS1 protein levels by western blot.

Previous studies have shown that WWP1 usually causes the degradation of its target proteins through ubiquitin-mediated proteasome pathway [18]. To further confirm whether WWP1 negatively regulates LATS1 through its ubiquitin ligase activity, we first constructed a plasmid expressing a ligase-dead WWP1 (WWP1-C890A-myc) and tested its ability to degrade LATS1. Importantly, WWP1-C890A was unable to degrade LATS1 even at the highest concentration (Fig. 4A). In addition, transfection of increasing amounts of WWP1 plasmid into cells has no effect on Ndr1, a LATS1 homolog lacking PPxY motifs at the N-terminal domain of LATS1 (Fig. 4B). Moreover, over-expression of WWP1 in MCF10A immortalized mammary cells caused reduced levels of endogenous LATS1 (Fig. 4C). Most importantly, reduced level of LATS1 by WWP1 is indeed at the protein level. When protein synthesis is blocked by CHX, over-expression of WWP1 causes increased protein degradation (Fig. 4D). qRT-PCR analysis also shows that over-expression of even 18-fold WWP1 mRNA has no significant effect on the levels of LATS1 mRNA (Fig. 4E). Finally, we provide evidence that WWP1 rather than its ligase-dead (WWP1-C890A) or WW domain-lacking (WWP1-ΔWW) mutant is able to degrade LATS1 through ubiquitination both *in vivo* and *in vitro* and that the 26S proteasome inhibitor MG132 inhibits WWP1-induced LATS1 degradation (Fig. 5A–C, Fig. S1). In conclusion, our studies strongly suggest that WWP1 E3 ligase causes decreased LATS1 levels by causing protein degradation through ubiquitination and the proteasome-mediated pathway.

### WWP1 regulates breast cancer cell proliferation through down-regulation of LATS1

**Figure 6:**
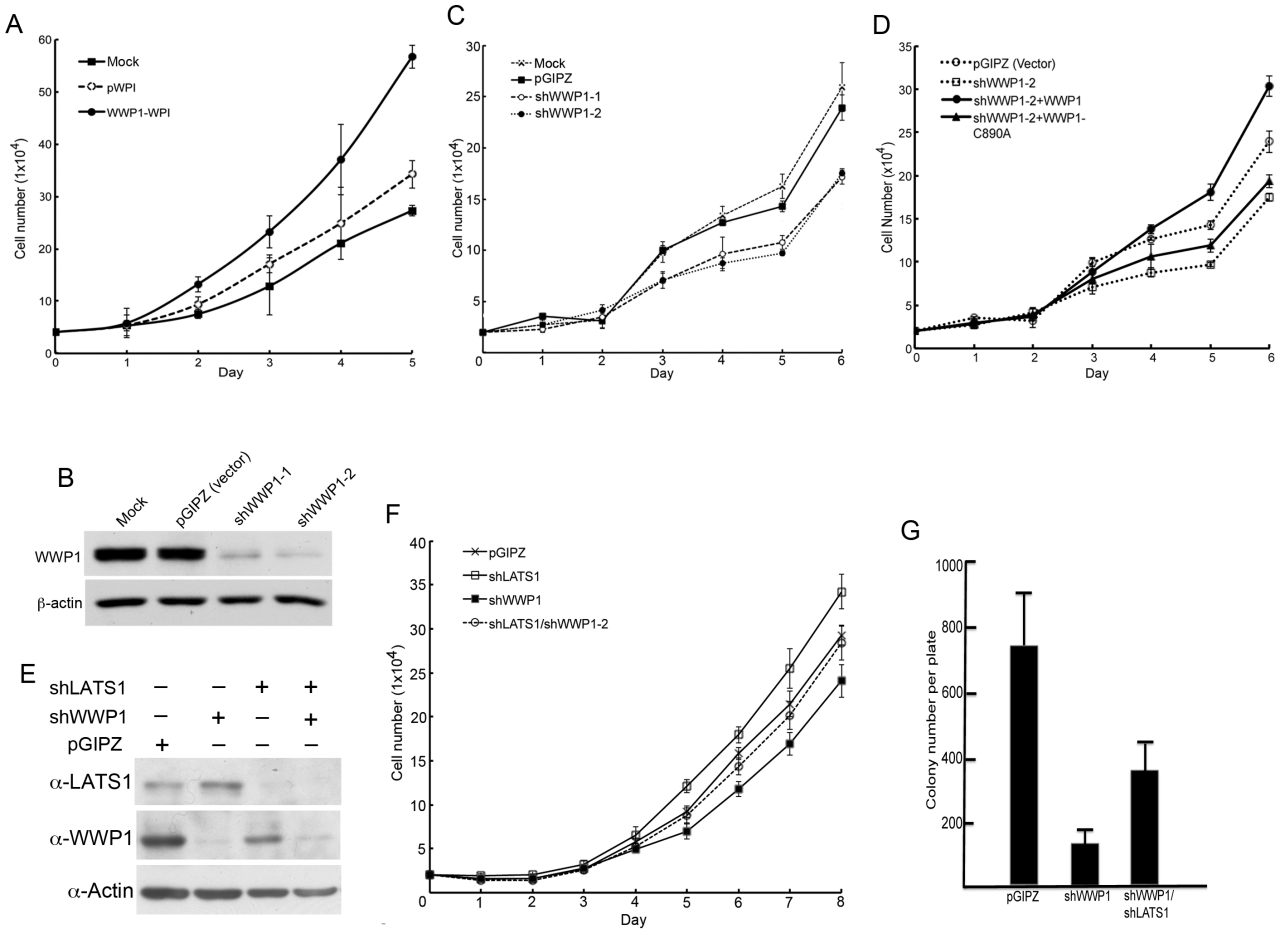
LATS1 mediates the effect of WWP1 on cell proliferation. A) Increased cell proliferation after WWP1 over-expression in MCF10A cells. Triplicate of 2×10^4^ infected MCF10A cells (mock) or MCF10A cells expressing WPI (vector) or WWP1-WPI were plated into each well of 24-well plate, followed by cell count for 5 days. The mean and standard deviation (SD) of three samples for each day are shown. B, C) Knockdown of WWP1 reduces cell proliferation assay. MCF7 cells were mock-infected or stably infected with lentivirus expressing pGIPZ (vector) or two shWWP1 shRNAs (shWWP1-1 and shWWP1-2). The levels of WWP1 and β-actin were examined by western blot (B). Cell proliferation analysis for these lines was performed for 6 days. The mean and standard deviation of three samples for each day are shown (C). D) Rescue of shWWP1-induced reduced cell proliferation by wild-type rather than ligase dead (WWP1-C890A). Cell proliferation was performed as described in (B). E, F) Knockdown of LATS1 rescues shWWP1-induced reduced cell proliferation. MCF7 cells were infected with lentivirus expressing pGIPZ (vector), shLATS1, shWWP1-1, and shWWP1-1/shLATS1. The levels of LATS1, WWP1, and β-actin were examined by western blot (E). Cell proliferation analysis was carried out for 8 days. The mean and standard deviation of three samples for each day are shown (F). G) Colony forming assay. About 1000 cells were plated in each of 100 mm plate, followed by incubation the cells at 37°C for 10 days. The mean and standard deviation of colony numbers from three plates for each cell line are shown.

In breast cancer, it has been shown that WWP1 is an oncogene and LATS1 is a tumor suppressor gene [3], [35], [38]. Therefore, WWP1 may induce tumorigenesis through down-regulation of LATS1. One of the key functions for an oncogene is its ability to induce increased cell proliferation. Consistent with previous studies [24], over-expression of WWP1 caused enhanced cell proliferation in LATS1-positive MCF10A mammary epithelial cells (Fig.4C and Fig. 6A), whereas knockdown of WWP1 by short-hairpin (sh) RNA (shWWP1-1 and shWWP1-2) in MCF7 breast cancer cells expressing functional wild-type LATS1 [16] significantly inhibited cell proliferation (Fig. 6B and 6C). In addition, introduction of wild-type WWP1 rather than ligase-dead WWP1 (WWP1-C890A) cDNA into MCF7 cells expressing shWWP1-2 (targeting the non-coding region of WWP1) rescued decreased cell proliferation induced by shWWP1 (Fig. 6D), confirming that reduced cell proliferation is due to down-regulation of WWP1 by shWWP1. To elucidate whether LATS1 functions downstream of WWP1 in regulating cell proliferation, we examined whether activation of LATS1 is responsible for the reduced cell proliferation after WWP1 knockdown by shRNA. Significantly, knockdown of LATS1 in MCF7 cells expressing shWWP1 rescued the reduced cell proliferation phenotype induced by WWP1 knockdown (shWWP1)(Fig. 6E–F). Similar results were obtained when colony forming assays were performed (Fig. 6G). These results strongly suggest that increased level of LATS1 may be responsible for reduced cell proliferation after WWP1 knockdown.

## Discussion

LATS1 is a ser/thr kinase and novel tumor suppressor down-regulated in various human cancers. Recently, LATS1 has been identified as a central player of a novel Hippo signaling pathway that plays essential roles in organ size control, tumorigenesis, stem cell differentiation/renewal, etc. Although the function of LATS1 in tumorigenesis has been extensively studied, how the level of LATS1 is controlled at the molecular level remains largely unknown. Recently, we have identified Itch ubiquitin ligase as the first negative regulator of LATS1 [16]. However, whether other ubiquitin ligases in the same family also have an effect on LATS1 is unknown. In addition, although LATS1 has been found down-regulated in breast cancer, how LATS1 interacts with other proteins in mammary tumorigenesis remains elusive. By screening members of the NEDD4-like family ubiquitin ligases that play important roles in cancer, we have identified WWP1 as a novel negative regulator of LATS1 tumor suppressor stability. We have also shown that WWP1 regulates breast cancer cell proliferation by down-regulating LATS1. WWP1 is an ubiquitin ligase responsible for regulating the protein stabilities of many proteins such as p63, Smad, and ErbB2, etc [18]. However, none of them has been shown to be responsible for WWP1-induced increased cell proliferation. In addition, our results suggest that in addition to mRNA down-regulation by promoter hypermethylation [35], down-regulation of LATS1 at the protein levels through WWP1-mediated ubiquitination and degradation may be a novel mechanism by which LATS1 is down-regulated in various cancers. Further examining the correlation between levels of LATS1 and WWP1 using clinical breast and prostate cancer may provide further *in vivo* data regarding how dysregulation of these two proteins are involved in the development of human cancers.

Although all of the NEDD4-like family member ligases can induce dose-dependent degradation of LATS1 when over-expressed (Fig. 1A), only loss of endogenous Itch and WWP1 causes increased LATS1 protein stability (Fig. 1B). This suggests that only Itch and WWP1 are essential to maintain the steady-state stability of LATS1. Under normal physiological conditions, protein level is dynamically controlled by its synthesis and degradation. Ubiquitin-mediated protein degradation is the most commonly used system in cells to induce protein degradation. From our studies, WWP1 and Itch serve to balance LATS1 tumor suppressor activity through continuous ubiquitination and degradation of LATS1 under physiological (unstressed) condition. It is unclear why LATS1 needs two ubiquitin ligases to regulate its stability. However, previous studies have shown that several ubiquitin ligases can regulate the same substrate under different physiological conditions or cell types. For example, p53 is regulated by 15 ubiquitin ligases [39]. The stability of p53 can be negatively regulated by both Mdm2 and Pirh2 kinases. However, Mdm2 can cause p53 degradation under unstressed condition [40], whereas Pirh2 rather than Mdm2 can be activated to degrade p53 upon DNA damage [41]. In addition, our data suggest that WWP1 and Itch may have overlapping functions in the regulation of LATS1 stability. Therefore, knockdown of either WWP1 or Itch alone will partially cause imbalance of LATS1 production and degradation, resulting in enhanced levels of LATS1. Similar results, in which knockdown of either WWP1 or Itch causes enhanced levels of ErbB4, were also reported by others [22]. Moreover, Pirh2 is up-regulated in lung cancer and affects lung tumorigenesis by reducing p53 activity [42], whereas Mdm2 is amplified in sarcomas [43]. Consistent with this notion, WWP1 rather than Itch is amplified and up-regulated in breast cancer [25]. Therefore, it is possible WWP1 and Itch regulates LATS1 stability in different cancer types and physiological conditions.

Previous studies have shown that WW domains 1 and 3 of WWP1 are type I WW domains that bind to PPxY motifs with higher affinities as compared to WW domains 2 and 4 [22], [44]. However, our studies showed that LATS1 binds to the WW domains 1–3 of WWP1 through its two PPxY^376^ and PPxY^559^ motifs. The structural interaction between the 3 WW domains and 2 PPxY motifs is currently unknown. Interestingly, previous studies also showed that LATS1 suppresses tumor cell growth by interacting with two WW domains of its substrates YAP and TAZ using its PPxY motifs [9], [10], [12], [37], [45]. It is therefore possible that WWP1, with four WW domains, may compete with YAP and TAZ for LATS1 binding to control the levels of tumor suppressor LATS1. Since loss of WWP1 causes enhanced levels of LATS1 and decreased tumor cell growth, development of strategies that specifically target WWP1 or disrupt LATS1 and WWP1 interaction in breast cancers to activate LATS1 may be a useful approach for successful cancer therapy.

In conclusion, our study has identified WWP1 E3 ligase as a novel negative regulator of LATS1 tumor suppressor stability. Further characterization of their functional interactions in mice and examination of their correlations in clinical breast cancer patients will provide useful information for future targeting of WWP1-LATS1 interaction in the treatment of breast cancer.

## Supporting Information

Figure S1 ***In vitro*** **ubiquitination of LATS1 and its mutants.** About 500 µg of protein lysate expressing LATS1-myc or LATS1-Y376A/Y559A-myc were immunoprecipitated with 2 µg of anti-LATS1 mAb (Cell Signaling). The precipitated proteins were used for *in vitro* kinase assay. The experimental procedures were as described in legend of Fig. 5B.
(TIF)
