# Supplementary figures and images for: WWP1 E3 Ligase Targets LATS1 for Ubiquitin-Mediated Degradation in Breast Cancer Cells
Source: PLoS One. 2013 Apr 3;8(4):e61027. doi: 10.1371/journal.pone.0061027 (PMC3616014; doi:10.1371/journal.pone.0061027)

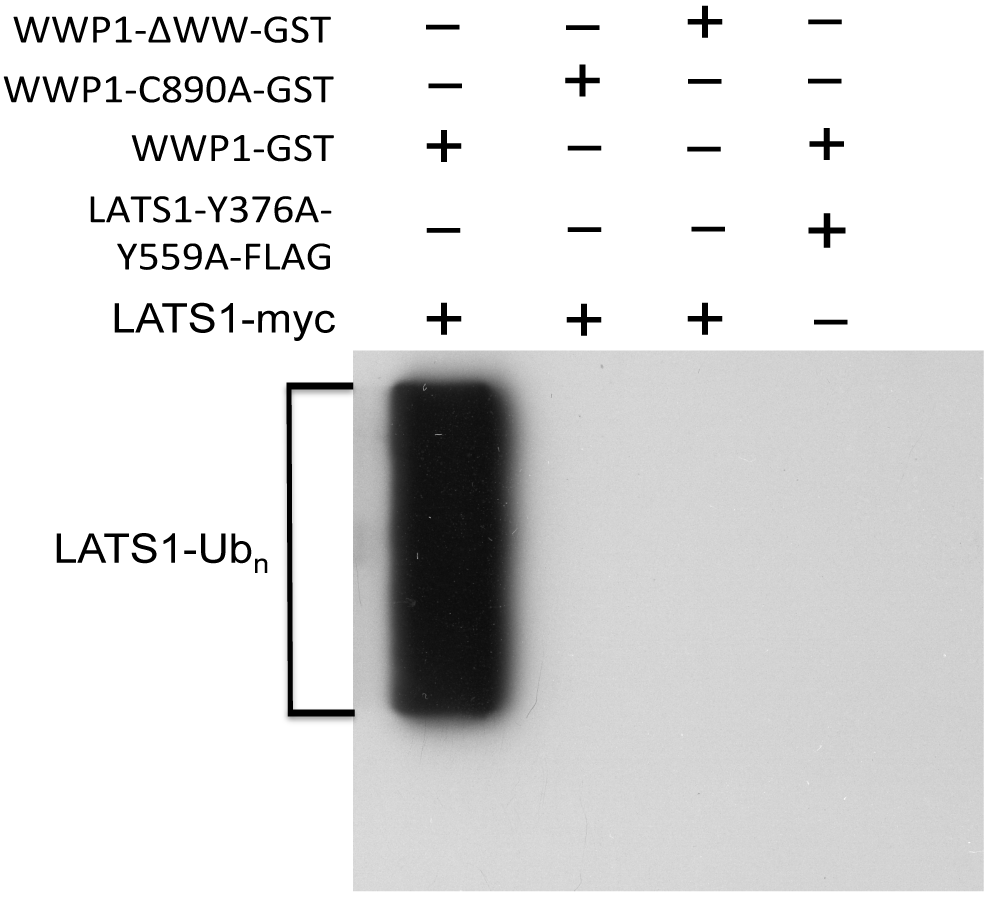

Supplement: Figure S1 — In vitro ubiquitination of LATS1 and its mutants. About 500 µg of protein lysate expressing LATS1-myc or LATS1-Y376A/Y559A-myc were immunoprecipitated with 2 µg of anti-LATS1 mAb (Cell Signaling). The precipitated proteins were used for in vitro kinase assay. The experimental procedures were as described in legend of Fig. 5B. (TIF) [file pone.0061027.s001.tif]
